# Quality of Life in Patients With Primary and Metastatic Brain Tumors in the Literature as Assessed by the FACT-Br

**DOI:** 10.4021/wjon585w

**Published:** 2013-01-04

**Authors:** Nicholas Chiu, Leonard Chiu, Liang Zeng, Liying Zhang, David Cella, Marko Popovic, Ronald Chow, Henry Lam, Michael Poon, Edward Chow

**Affiliations:** a Rapid Response Radiotherapy Program, Department of Radiation Oncology, Odette Cancer Centre, Sunnybrook Health Sciences Centre, University of Toronto, Toronto, Ontario, Canada; b Department of Medical Social Sciences, Northwestern University Feinberg School of Medicine, Chicago, IL, USA

**Keywords:** FACT-Br, Quality of Life, Brain metastases, Primary brain tumors

## Abstract

**Background:**

The Functional Assessment of Cancer Therapy-Brain (FACT-Br) is a quality of life (QOL) assessment tool that was originally developed for use in patients with primary brain tumors. However, the tool has also been used to assess QOL in patients with metastatic brain tumors. The purpose of this study is to compare the differences in QOL responses as assessed by the FACT-Br in patients with primary and metastatic brain neoplasms.

**Methods:**

A systematic literature search was conducted using the OvidSP platform in MEDLINE (1946 to July Week 2 2012) and EMBASE (1980 to 2012 Week 28). Articles in which the FACT-Br was used as a QOL assessment for patients with malignant brain tumors (both primary and metastatic) were included in the study. The weighted means of FACT-Br subscale and overall scores were calculated for the studies. To compare these scores, weighted analysis of variance was conducted and PROC GLM was performed for the data. A P-value of < 0.05 was considered statistically significant.

**Results:**

A total of 23 studies (four in brain metastases, 18 in primary brain tumors and 1 in a mixed sample) using the FACT-Br for assessment of QOL were identified. Social and functional well-being were significantly better in patients with primary brain tumors (weighted mean score of 22.2 vs. 10.7, P = 0.0026, 16.9 vs. 6.2, P = 0.0025, respectively). No other scale of the FACT-Br was significantly different between the two groups and the performance status of patients included in both groups was similar.

**Conclusion:**

Patients with primary brain cancer seemed to have better social and functional well-being scores than those with metastatic brain tumors. Other QOL domains were similar between these two groups. However, the heterogeneity in the included studies and the low sample size of included samples in patients with metastatic brain tumors could have confounded our findings.

## Introduction

Malignant brain tumors can be broadly divided into primary brain tumors (namely tumours originating in the brain) and secondary brain tumors (namely brain metastases). Both cause significant morbidity for affected individuals. These patients typically have very short life expectancies: in metastatic brain tumor patients, the median survival has been found to be within several months [1]; in primary brain tumor patients, median survival is not much better, typically within a few months or years [2]. Patients with metastatic brain tumors also face burden from other possible systemic diseases. As the prognosis is extremely poor, palliation, rather than cure, is often the more suitable treatment for this patient population. In many cases, management and prevention of complications are the targets of treatment. As such, quality of life (QOL) is an important consideration.

QOL is a subjective, multidimensional construct that focuses on several key domains that emphasize a patient’s wellbeing [3]. As QOL reflects a patient’s individual situation, it is most commonly assessed through the use of self-reported questionnaires completed by the patient.

The Functional Assessment of Cancer Therapy (FACT) group offers questionnaires developed for the assessment of QOL in cancer patients. In addition to the FACT-General (FACT-G), a core questionnaire used to determine the more general domains of QOL among all cancer patients, other disease-specific questionnaires are also available. The FACT-Brain (FACT-Br) is one such instrument that assesses brain-tumor related QOL issues. This tool consists of the FACT-G plus a brain-tumor specific scale. A total of 50 items are included that cover the following domains of QOL: physical well-being, social/family well-being, emotional well-being, functional well-being, and disease specific concerns. Patients are asked to indicate the presence/severity of certain issues/symptoms on a scale of 0 - 4 (a 5-point Likert Scale).

While the FACT-Br was originally developed for use in patients with primary brain tumors, the tool has also been used to assess QOL in patients with metastatic brain tumors. How patients with primary brain tumors and patients with metastatic tumors respond to the FACT-Br may be of interest to health-care professionals in understanding the potential differences between QOL in the two patient populations.

The purpose of this study is to compare and evaluate the difference in FACT-Br QOL responses between patients with primary brain tumors and metastatic brain tumors as reported in the literature.

## Methods and Materials

A systematic literature search was conducted over the OvidSP platform in MEDLINE (1946 to July Week 2 2012) and EMBASE (1980 to 2012 Week 28). The following search terms were used in a variety of combinations: FACT-Br, FACT-Brain, Functional Assessment of Cancer Therapy, FACT, primary tumor, brain metastases, palliative, quality of life, questionnaire, and instrument assessment. No restrictions were made on language.

Articles in which the FACT-Br was used as a QOL assessment for patients with malignant brain tumors (both primary and metastatic) were included in the study. Studies were only included if prospective data from the FACT-Br were available. Primary outcomes of interest were FACT-Br scores and demographic parameters of patients included in the studies (namely gender, primary cancers, median age, etc.). Although articles in which patients’ baseline subscale scores for the FACT-Br were of primary interest, articles that only included the overall FACT-Br score were also included in the study. Reference lists of extracted studies were explored along with lists of other publications that cited the reference. Articles were identified and data were extracted independently by five authors.

**Figure 1:**
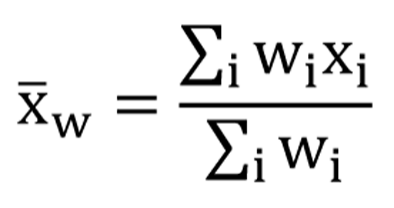
The weighted mean.

**Figure 2:**
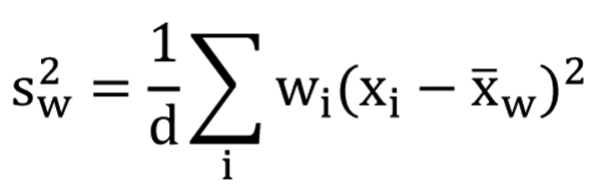
The weighted variance.

The scores of the FACT-Br and demographic data were extracted for all included studies. To compare the median KPS and average FACT-Br scales in patients with primary and metastatic brain tumors, weighted analysis of variance (ANOVA), based on the number of patients in the QOL data reported, was conducted and PROC GLM was performed for the data. To normalize the distribution of KPS and FACT-Br scales, natural log-transformation was applied. The weighted arithmetic means and the weighted standard deviation (SD) of the FACT-Br were also calculated in patients with primary and metastatic brain tumors. The weighted mean was defined as in Figure 1, and the weighted variance was defined as in Figure 2, where w*_i_* is the weight for the *i*th strudy, x*_i_* is the *i*th variable value, and the divisor d is n-1. The weighted variance is a measure of variability, and it is the sum of the weighted squared distance of data values from the mean divided by the variance divisor which is defined to be n-1. A P-value of < 0.05 was considered statistical significant. All analyses were conducted using Statistical Analysis of Software (SAS version 9.2 for Windows).

## Results

A total of 635 publications were identified in the search. Of those, a total of 23 studies using the FACT-Br for assessment of QOL in patients with malignant brain tumors were identified with reported QOL results [2, 4-25]. Of the 23 studies (29 arms in total), 4 studies utilized the FACT-Br as an assessment tool for QOL in patients with brain metastases [4, 12, 18, 24]; one of the identified studies used the FACT-Br for both primary and metastatic patients [13]; and the remaining 18 studies composed of patients with primary brain cancer[2, 5-11, 14-17, 19-23, 25].

The FACT-Br was published in 1995 by Michael A. Weitzner et al [20]. All the studies using FACT-Br were published afterwards with sample sizes ranging from 7 to 718. The included studies came from medical centers across 5 different countries: 16 publications from the United States [6-9, 11-20, 22, 23], 3 from Canada [2, 4, 25], 2 from Italy [5, 10], and 1 each from China [24] and Japan [21]. Butler et al and Roger et al [13, 18] were excluded from analysis as the former included patients with both primary and metastatic brain tumors and the latter only reported the median FACT-Br score, rather than the mean.

### Demographics and clinical characteristics

While the majority of the studies included did not disclose whether their patients were in-patients or out-patients, 4 of the identified studies indicated that their patient sample was entirely composed of in-patients [5, 11, 17, 19] only one study explicitly indicated an exclusively out-patient sample (Table 1) [20].

**Table 1:** Included Studies

Author	Year	Type	No. of patients	In/Outpatient	PWB	SWB	EWB	FWB	Concerns	Overall	KPS MED
Weitzner [20]	1995	PRIMARY	101	Outpatient	22.25	21.73	15.96	19.92	n/a	n/a	n/a
Sutton [17]	1999	PRIMARY	22	Inpatient	24	24	17	24	n/a	n/a	n/a
Huang [19]	2001	PRIMARY	10	Inpatient	n/a	n/a	n/a	n/a	n/a	103	n/a
Pelletier [2]	2002	PRIMARY	60	n/a	n/a	n/a	15.6	n/a	n/a	136.3	n/a
Roa (1st arm) [25]	2004	PRIMARY	47	n/a	n/a	n/a	n/a	n/a	n/a	75.1	70
Roa (2nd arm) [25]	2004	PRIMARY	48	n/a	n/a	n/a	n/a	n/a	n/a	77.7	70
Brown [22]	2006	PRIMARY	190	n/a	n/a	n/a	n/a	n/a	n/a	62	n/a
Shaw [9]	2006	PRIMARY	34	n/a	20.7	20.2	17.9	18	47.6	124.4	n/a
Locke [7]	2007	PRIMARY	205	n/a	81.3	n/a	72.4	n/a	69.2	72.8	n/a
Locke (1st arm) [8]	2008	PRIMARY	7	n/a	n/a	n/a	n/a	n/a	n/a	132	n/a
Locke (2nd arm) [8]	2008	PRIMARY	12	n/a	n/a	n/a	n/a	n/a	n/a	117	n/a
Jones [16]	2009	PRIMARY	171	n/a	22	23	19	19	50	133	90
Kvale [11]	2009	PRIMARY	50	Inpatient	21.2	23.3	19	15.1	48.8	127.33	n/a
Liu [23]	2009	PRIMARY	65	n/a	23	24.1	17.7	18.2	n/a	n/a	90
Mobed [15]	2009	PRIMARY	718	n/a	20.9	22.7	17.4	16.4	51.6	129	90
Lucchiari (1st arm) [5]	2010	PRIMARY	27	Inpatient	21.62	21.69	20.62	13.99	49.16	127.08	n/a
Lucchiari (2nd arm) [5]	2010	PRIMARY	29	Inpatient	20.73	17.69	17.69	14.13	47.48	117.72	n/a
Lucchiari (3rd arm) [5]	2010	PRIMARY	28	Inpatient	21.94	16.77	17.27	14.17	53	123.15	n/a
Lamperti [5]	2011	PRIMARY	81	n/a	22.92	18.54	17.86	14.15	n/a	n/a	n/a
Ruiz [14]	2011	PRIMARY	18	n/a	21	24.1	18.5	16.5	47.1	127.2	n/a
Terasaki [21]	2011	PRIMARY	26	n/a	n/a	n/a	n/a	n/a	n/a	n/a	60
Bezjak [4]	2002	METASTATIC	75	n/a	n/a	n/a	n/a	n/a	n/a	123.1	n/a
Rogers [18]	2006	METASTATIC	71	n/a	n/a	n/a	n/a	n/a	n/a	n/a	90
Chang (1st Arm) [12]	2009	METASTATIC	30	n/a	n/a	n/a	n/a	n/a	n/a	64.6	70
Chang (2nd Arm) [12]	2009	METASTATIC	28	n/a	n/a	n/a	n/a	n/a	n/a	59.8	80
Ma [24]	2009	METASTATIC	21	n/a	13.8	10.7	12.1	6.2	26.6	69.1	n/a

PWB: physical well-being; SWB: social well-being; EWB: emotional well-being; FWB: functional well-being; KPS: Karnofsky Performance Status; MED: median; n/a: not available.

Of the 29 study arms included in the review, 5 included a sample composed of 40.0% to 49.9% male patients [4-6, 12, 24], 11 included a sample composed of 50.0% to 59.9% male patients [2, 5, 8, 9, 11, 13, 15, 20, 25], 11 included a sample composed of 60.0% to 69.9% [5, 7, 21-26, 27], and 2 included a sample composed of 70.0-79.9% male patients [10, 17].

Mean age was reported in 18 of 29 of the arms while median age was reported in 16 (some reported both). The majority of studies included patients equal to or greater than 40 years of mean age [2, 5-8, 10, 11, 15, 16, 19, 20, 22, 25]. In studies that reported the median age, all included patients equal to or greater than 40 years of age [5, 7-9, 12-15, 18, 21-23].

### Weighted means

The Median KPS and average FACT-Br scales are shown in Table 1. Weighted means and P-values were calculated for KPS and FACT-Br scales in patients from primary or metastatic brain tumor groups (Table 2).

**Table 2:** Weighted Means of KPS and FACT-Br Subscale Scores

	Primary Brain Tumors		Metastatic Brain Tumor		P-value†
	Sum of Weight	Weighted Mean (SD)	Sum of Weight	Weighted Mean (SD)	
KPS	1,075	87.51 (104.61)	129	83.18 (66.32)	0.6631
Physical well-being	1,549	29.38 (230.56)	21	13.8 (NA*)	0.5952
Social well-being	1,344	22.23 (17.54)	21	10.7 (NA)	0.0026
Emotional well-being	1,609	24.58 (203.53)	21	12.10 (NA)	0.6412
Functional well-being	1,344	16.92 (20.34)	21	6.20 (NA)	0.0025
Brain subscale: Concerns related to Brain Metastasis	1,280	53.81 (86.37)	21	26.60 (NA)	0.0619
Overall Scale	1,654	111.27 (290.24)	154	92.83 (212.13)	0.4386

* NA: not available because there was only one study with available subscales; †: P-value was obtained by weighted analysis of variance; natural log-transformation was applied for KPS, FACT-Br subscales, and overall scale.

For primary brain tumor patients, the weighted mean of their median KPS scores was 87.51; in comparison, for metastatic brain tumor patients, the weighted mean of their median KPS scores was 83.18. The difference was not statistically significant (P = 0.66).

Unfortunately, while 3 included studies (totalling a combined sample size of 154 patients) provided overall FACT-G scores [4, 12, 24], only one of them included subscale score data for the FACT-Br [24].

The mean overall FACT-Br (FACT-G +Br Subscale) score in patients with primary brain tumors was 111.27 [2, 5, 7, 8, 11, 14-16, 19, 22, 25]. In comparison, the mean overall FACT-Br score in patients with metastatic brain tumors was 92.83 [4, 12, 24]. However, this difference was not statistically significant (P = 0.44). For the brain subscale, the mean subscale score in patients with primary brain tumors was 53.81 [9, 11, 14, 15] whereas that in patients with metastatic brain tumors was 26.60 [24]. Again, this difference was not statistically significant (P = 0.062) (Table 2).

Patients with primary brain tumors generally had worse QOL as assessed by the FACT-G component. Physical well-being, social/family well-being, emotional well-being, and functional well-being for primary vs. metastatic patients were: 29.38 vs. 13.60, 22.23 vs. 10.70, 24.58 vs. 12.10, and 16.92 vs. 6.20 respectively.

There was a statistical difference in the social and functional well-being scales between patients with primary and metastatic brain tumors (P = 0.0026, and P = 0.0025) (Table 2). This suggests that patients with metastatic brain tumors were more likely to have a statistically significant worsening in social and functional well-being subscale scores compared to those with primary brain tumors. There was no statistical significant difference in median KPS and other FACT-Br subscales between patients with primary and metastatic brain tumors.

## Discussion

A trend was noticed in the differences between metastatic patient responses vs. primary patient responses on the FACT-Br. Metastatic patient response scores on the FACT-Br seemed to be lower on all 4 wellbeing subscales as well as the additional brain subscale and the overall FACT-Br score - seemingly suggesting a relatively better QOL for primary brain tumor patients. A statistical difference in the social and functional well-being scales between patients with primary and metastatic brain tumors was found.

To our knowledge, no studies have exclusively observed the difference in social and functional well-being in patients suffering from primary brain cancer compared to patients suffering from metastatic brain cancer. On the other hand, several studies have compared QOL between the two groups based on the stage of disease. Siddiqi et al investigated the difference in QOL between the primary and metastatic cancer patients in general [27]. They observed the differences in QOL (symptom severity and physical functioning) experienced by primary non-metastatic (PNM), primary metastatic (PM) and recurrent (RC) cancer patients. RC cancer patients reported the worst symptom severity and physical function followed by PM and PNM patients. These findings are consistent with our results, as the authors observed QOL to be generally worse in metastatic patients compared with primary patients: an observation that holds true for the current study as well.

An important limitation of our study is that only 5 studies used the FACT-Br as a QOL assessment tool in brain metastases patients; in addition, only one study actually included the individual subscale scores of the FACT-Br responses with a sample size of 21 patients. Thus the weighted average of individual subscale responses to the FACT-Br in metastatic brain tumor patients is solely based on patients’ responses from this one study. With validation and continued adoption, future studies should continue to assess patients with brain metastases using the FACT-Br. KPS was not a confounding variable in this study because the difference in the weighted means of the median KPS scores obtained in the two patient populations was not found to be statistically significant.

Another limitation was the heterogeneity of the studies and patients included. Because the disease progression of patients in each study was not known entirely, progression could have varied greatly from patient to patient and from study to study. Such differences in disease progression could explain differences in QOL. For example, if brain tumor patients in all the primary cancer studies were further along in disease progression than the metastatic patients, a difference in QOL would, naturally, be expected.

The results of this study pave the way for possible courses of action in identifying more certainty in this field. Several recommendations for future research in the field include: first, more studies like the ones done by Chang et al [12], Ma et al [24], and Bezjak et al [4] should be done using the FACT-Br as a QOL assessment tool in metastatic patients: as not many were found to exist in the databases included in the literature search. As more studies utilizing the FACT-Br in the metastatic patient population are conducted, the current study could be repeated to confirm the validity and accuracy of our results. More importantly, there is a need to validate the FACT-Br in the metastatic patient population.

### Conclusion

Patients with primary brain cancer seem to have better social and functional well-being than those with metastatic brain tumors. Other QOL domains are similar between these two groups. The readers are cautioned of the limitations of our study.
